# Protective Coupling of Mitochondrial Function and Protein Synthesis via the eIF2α Kinase GCN-2

**DOI:** 10.1371/journal.pgen.1002760

**Published:** 2012-06-14

**Authors:** Brooke M. Baker, Amrita M. Nargund, Tiffany Sun, Cole M. Haynes

**Affiliations:** 1 Cell Biology Program, Memorial Sloan-Kettering Cancer Center, New York, New York, United States of America; 2 BCMB Allied Program, Weill Cornell Medical College, New York, New York, United States of America; Max Planck Institute for Biology of Aging, Germany

## Abstract

Cells respond to defects in mitochondrial function by activating signaling pathways that restore homeostasis. The mitochondrial peptide exporter HAF-1 and the bZip transcription factor ATFS-1 represent one stress response pathway that regulates the transcription of mitochondrial chaperone genes during mitochondrial dysfunction. Here, we report that GCN-2, an eIF2α kinase that modulates cytosolic protein synthesis, functions in a complementary pathway to that of HAF-1 and ATFS-1. During mitochondrial dysfunction, GCN-2–dependent eIF2α phosphorylation is required for development as well as the lifespan extension observed in *Caenorhabditis elegans*. Reactive oxygen species (ROS) generated from dysfunctional mitochondria are required for GCN-2–dependent eIF2α phosphorylation but not ATFS-1 activation. Simultaneous deletion of ATFS-1 and GCN-2 compounds the developmental defects associated with mitochondrial stress, while stressed animals lacking GCN-2 display a greater dependence on ATFS-1 and stronger induction of mitochondrial chaperone genes. These findings are consistent with translational control and stress-dependent chaperone induction acting in complementary arms of the UPR^mt^.

## Author Summary

Defects in mitochondrial function are associated with numerous age-related diseases including cancer and Parkinson's. Mitochondrial function relies upon maintenance of the mitochondrial proteome, which is comprised of nuclear and mitochondrial-encoded proteins. Nuclear-encoded polypeptides are translated in the cytosol and must be transported into the mitochondrial matrix, where resident chaperones facilitate folding into their functional conformation. In order to protect against dysfunction arising from an accumulation of misfolded or unfolded mitochondrial proteins, cells employ mechanisms to maintain the folding environment. One such signaling pathway is mediated by the bZip transcription factor ATFS-1, which upregulates mitochondrial chaperones to accommodate an overwhelming misfolded protein load. Here, we describe a complementary pathway that couples the mitochondrial functional status with the rate of cytosolic protein synthesis to protect the organelle from incoming unfolded protein substrates during mitochondrial stress. This pathway is regulated by the cytosolic kinase GCN-2, which phosphorylates the translation initiation factor 2α (eIF2α) subunit to slow general translation. GCN-2 responds to ROS emitted from dysfunctional mitochondria to promote growth and extend lifespan during mitochondrial stress.

## Introduction

Mitochondrial dysfunction and altered protein homeostasis are associated with numerous developmental and age-related diseases as well as the general process of aging [1]. The mitochondrial protein-folding environment is maintained by nuclear-encoded mitochondrial chaperones, which promote efficient protein folding, and proteases that degrade those proteins that fail to fold or oligomerize correctly [1], [2], [3]. Protein folding is compartmentalized in eukaryotic cells and facilitated by compartment-specific folding machinery in the cytosol, endoplasmic reticulum (ER) and mitochondria. As threats to protein homeostasis affect the folding compartments differently, each compartment has dedicated stress responses or unfolded protein response (UPR) signaling pathways to transcriptionally regulate organelle-specific molecular chaperones and reduce the protein-folding load on the resident protein folding machinery.

Dysfunction and accumulation of misfolded proteins in the ER triggers a multi-pronged unfolded protein response (UPR^ER^) that combines the upregulation of molecular chaperones to accommodate the folding requirements in the organelle with a reduction of cytosolic translation and ER protein import [4]. Activation of the transmembrane kinase PERK phosphorylates the cytosolic translation initiation factor eIF2α, thus attenuating general mRNA translation and reducing the load of incoming unfolded polypeptides [5]. In a complementary branch of the UPR^ER^, the transcription factor XBP-1 is activated and mediates the induction of ER-resident chaperones [6]. Thus, by coordinating signaling through parallel pathways, stress is relieved and organelle function restored. In contrast to these ER-protective mechanisms, signaling pathways that protect the mitochondrial protein-folding environment are only beginning to emerge.

Maintenance of mitochondrial metabolic function depends on the efficient assembly of the mitochondrial proteome, which is comprised of nuclear-encoded as well as mitochondrial-encoded polypeptides [7]. Those proteins encoded by the nucleus are translated in the cytosol and post-translationally imported into mitochondria in an unfolded or unstructured state where they interact with the network of mitochondria-resident molecular chaperones. Failure of mitochondrial proteins to properly fold or oligomerize can result in electron transport chain (ETC) defects and accumulation of ROS, which further impacts additional mitochondrial activities including metabolic function. In order to respond to mitochondrial-specific stresses caused by the accumulation of unfolded proteins, depletion of mtDNA, defects in respiration or altered ROS metabolism, mitochondria have evolved stress response pathways that upregulate mitochondrial molecular chaperones to restore organelle homeostasis [8], [9], [10]. One of these pathways, termed the mitochondrial unfolded protein response (UPR^mt^), couples the status of the mitochondrial protein-folding environment to the transcription of mitochondrial chaperone genes [8], [9]. The complement of nuclear-encoded mitochondrial chaperones, such as mtHsp70 and HSP-60, assist in import, folding, and assembly of multi-protein complexes in the matrix and on the matrix side of the inner mitochondrial membrane [2]. Increased levels of mitochondrial dysfunction perturb the balance between chaperones and their client proteins, leading to activation of the UPR^mt^ and upregulation of mitochondrial chaperone genes to re-establish homeostasis [8], [9], [10].

Our previous genetic studies in *C. elegans* have identified several proteins required for signaling the response including the mitochondrial inner membrane-localized peptide transporter HAF-1 and the bZip transcription factor ZC376.7 [11], which was recently renamed ATFS-1 (**A**ctivating **T**ranscription **F**actor associated with **S**tress-1). Mitochondrial dysfunction triggers the HAF-1-dependent nuclear accumulation of ATFS-1, resulting in the upregulation of mitochondrial chaperone genes including HSP-60 and mtHsp70. Activation of this pathway occurs in response to elevated levels of mitochondrial stress, which can be the result of accumulation of unfolded proteins beyond the capacity of mitochondrial molecular chaperones [8] as well as increased levels of oxidative stress [9], respiratory chain dysfunction and by mtDNA depletion [10]. Thus, this mitochondrial stress response pathway, although termed a UPR because of conceptual similarities with the XBP-1 branch of the UPR^ER^, responds to diverse insults to mitochondrial function.

In addition to chaperone induction, the UPR^ER^ also mediates the attenuation of cytosolic translation to protect the ER during stress. Similarly, inhibition of cytosolic translation has been suggested to promote mitochondrial function in yeast and *Drosophila* models of mitochondrial stress, although a potential regulatory mechanism(s) remained to be elucidated [12], [13]. Cytosolic translation attenuation via PERK-1-mediated eIF2α phosphorylation promotes ER function during stress by reducing the client load on ER-resident chaperones [5], [14]. Additionally, in *C. elegans* genetic manipulations that reduce cytosolic translation rates provide resistance to numerous stresses including heat shock and also extend lifespan [15], [16], [17]. Several signaling pathways are known to regulate translation rates in eukaryotic cells including TOR-regulated phosphorylation of S6 kinase and 4E-BP [16], [17], [18], however a mechanism to couple cytosolic translation rates to mitochondrial function has not been demonstrated.

Phosphorylation of eIF2α by four dedicated kinases (GCN2, PERK, HRI and PKR) serves to attenuate cytosolic translation in response to a variety of cellular stresses including starvation, oxidative stress, viral infection and unfolded protein stress in the ER [19], [20], . In yeast and mammals, GCN-2 phosphorylates eIF2α in response to conditions of low free amino acid levels and oxidative stress [22], [23]. Here we describe experiments demonstrating that in *C. elegans*, translation attenuation via GCN-2-dependent eIF2α phosphorylation acts in a responsive and adaptive protective pathway during mitochondrial stress to promote mitochondrial function. Phosphorylation levels of eIF2α are increased during mitochondrial stress, which requires ROS generated from dysfunctional mitochondria. Our data demonstrate that GCN-2-dependent translational control acts in a mitochondrial protective signaling pathway complementary to the regulation of mitochondrial chaperone gene expression mediated by HAF-1 and ATFS-1.

## Results

### Mitochondrial Chaperone Induction by ATFS-1 Is Required for Development during Mitochondrial Stress

**Figure 1:**
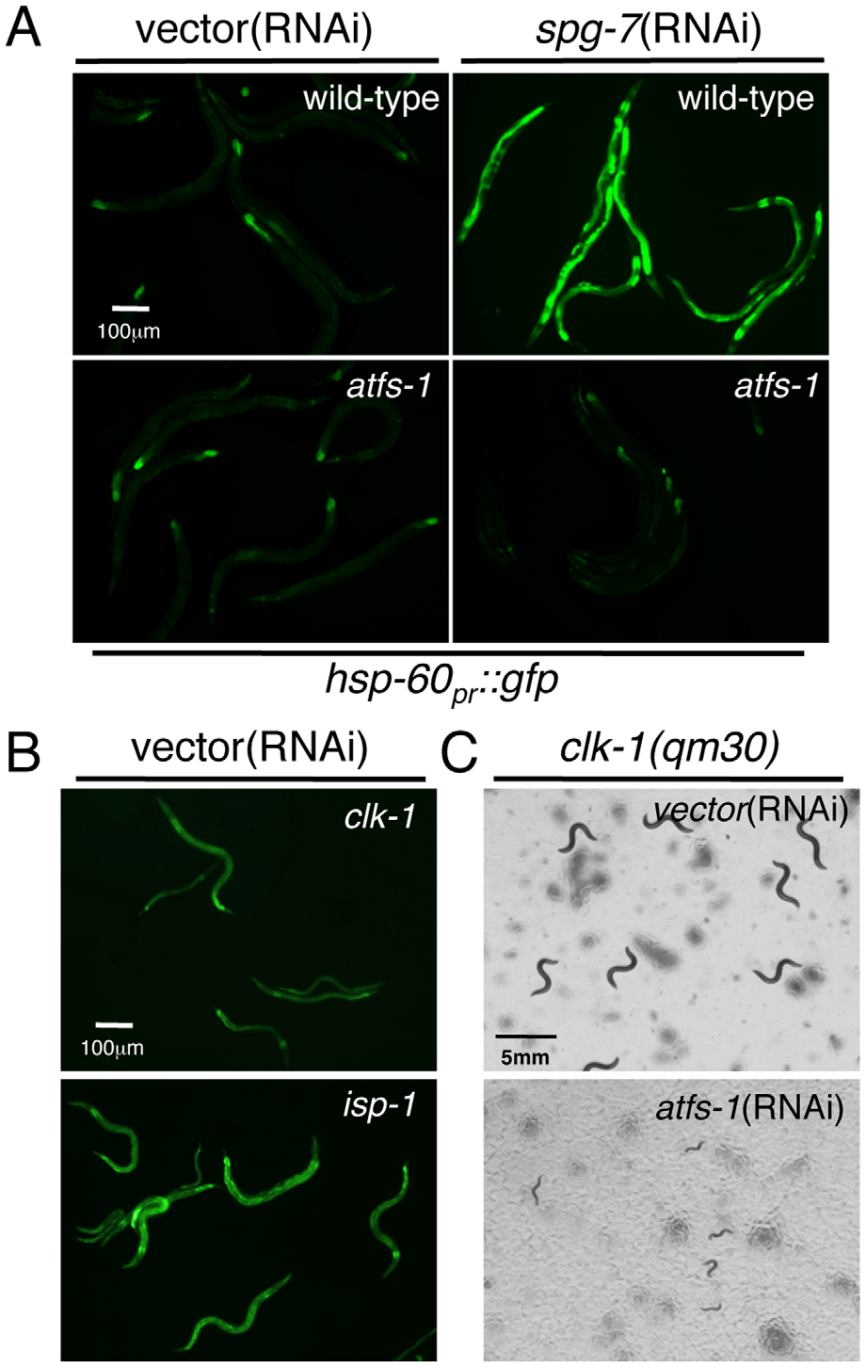
ATFS-1 Is Required for Mitochondrial Chaperone Induction and Development during Mitochondrial Stress. (A) Fluorescent photomicrographs of wild-type and *atfs-1(tm4525);hsp-60_pr_::gfp* transgenic worms raised on vector or *spg-7*(RNAi). (B) Representative fluorescent photomicrographs of *hsp-60_pr_::gfp* transgenic worms harboring the *clk-1(qm30)* or *isp-1(qm150)* alleles raised on vector(RNAi). (C) Images of *clk-1(qm30)* animals raised on vector or *atfs-1*(RNAi). Worms were plated at the L4 stage, allowed to develop to adulthood and lay eggs for 16 hours. The images were obtained five days after hatching.

We have previously described a mitochondrial stress response pathway that upregulates mitochondrial chaperone genes in response to multiple perturbations in mitochondrial function [8], [11], [24]. RNAi experiments indicated a requirement for the bZip transcription factor ATFS-1 in mitochondrial chaperone induction as demonstrated by quantitative PCR experiments as well as using reporter strains where the *hsp-60* promoter regulates expression of GFP (*hsp-60_pr_::gfp*) [11]. In order to corroborate the requirement for ATFS-1, we obtained the *atfs-1(tm4525)* deletion strain which lacks 432 base pairs and most of exons 2–4, and crossed it into the reporter strain. Unlike wild-type worms, *atfs-1(tm4525)* animals were unable to induce *hsp-60_pr_::gfp* when raised on *spg-7*(RNAi), a mitochondrial protease required for ETC quality control and mitochondrial ribosome biogenesis [25]. These results confirm the requirement for ATFS-1 in stress-induced mitochondrial chaperone gene induction (Figure 1A).

We next investigated ATFS-1-dependent *hsp-60_pr_::gfp* activation in strains harboring the well-characterized *clk-1(qm30)* or *isp-1(qm150)* mutations [26], [27]. *clk-1* encodes a mitochondrial protein required for ubiquinone synthesis [28], which acts as a lipid antioxidant throughout the cell and an electron transporter within the electron transport chain. *isp-1* encodes an iron-sulfur component of complex III in the ETC. As both mutations affect respiration and display impaired development [22], [23], we hypothesized that they would cause activation of the UPR^mt^. Indeed, *hsp-60_pr_::gfp* expression was consistently elevated in both strains consistent with the presence of mitochondrial stress. The *isp-1(qm150)* mutation caused considerably stronger *hsp-60_pr_::gfp* induction suggestive of a larger impact on mitochondrial function [29] (Figure 1B). Chaperone induction in both mutants required ATFS-1 as animals raised on *atfs-1*(RNAi) were unable to induce expression of *hsp-60_pr_::gfp* (data not shown).

To determine if the ATFS-1-dependent regulation of mitochondrial chaperone genes has a protective role during mitochondrial stress we examined the effect of *atfs-1*(RNAi) on the development of wild-type and mitochondrial stressed worms. As previously demonstrated, both *clk-1(qm30)* and *isp-1(qm150)* worms developed considerably slower than wild-type animals [22], [28]. Consistent with ATFS-1 being a stress responsive transcription factor, wild-type worms fed *atfs-1(RNAi)* developed at similar rates to wild-type animals (data not shown). However, feeding *clk-1(qm30)* and *isp-1(qm150)* worms *atfs-1*(RNAi) dramatically impaired their developmental rates (Figure 1C and Figure S1), indicating a requirement for ATFS-1 during development in the presence of mitochondrial stress.

### An RNAi Screen Suggests a Role for Translational Regulation in Protecting against Mitochondrial Stress

In addition to ATFS-1-regulated mitochondrial chaperone expression, we sought to identify additional components that promote mitochondrial protein homeostasis by acting in complementary pathways. To identify signaling pathways that act in parallel to ATFS-1 we generated and screened an RNAi sub-library consisting of all *C. elegans* kinases and phosphatases [30]. We took advantage of *hsp-60_pr_::gfp* activation as a sensitive readout for the status of mitochondrial function to identify signaling components that promoted or impaired mitochondrial protein homeostasis. The *clk-1(qm30)* strain was chosen for the RNAi screen as it displayed mild *hsp-60_pr_::gfp* induction, potentially allowing for the identification of candidates whose knockdown by RNAi either decreased or further increased *hsp-60_pr_::gfp* expression (Figure 1B). We hypothesized that RNAi knockdown of candidates that act in a complementary protective signaling pathway would show enhanced *hsp-60_pr_::gfp* activation in the presence of stress because of an increased substrate load on the mitochondrial protein folding machinery. Alternatively, if components exist whose knockdown somehow enhances the protein-folding capacity, then those RNAi may suppress *hsp-60_pr_::gfp* activation in the *clk-1(qm30)* background.

**Figure 2:**
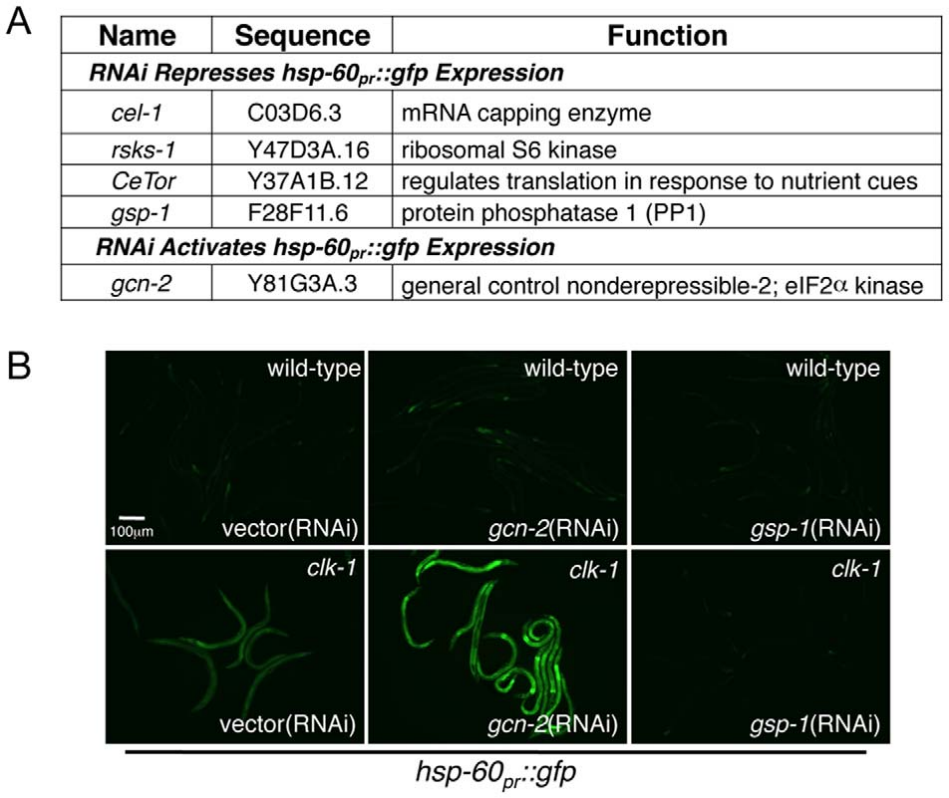
Identification of Kinases and Phosphatases That Affect Protein Synthesis Impact UPR^mt^ Activation. (A) Phosphatase and kinases whose knockdown by RNAi either increased or decreased *hsp-60_pr_::gfp* expression in *clk-1(qm30)* mutant worms. (B) Fluorescent photomicrographs of *hsp-60_pr_::gfp* expression in wild-type and *clk-1(qm30)* animals raised on vector, *gcn-2* or *gsp-1*(RNAi).

Interestingly, RNAi of several kinases required for protein synthesis reduced *hsp-60_pr_::gfp* expression in the *clk-1(qm30)* background (Figure 2A), which was also confirmed using the *isp-1(qm150)* strain (Figure S2A), suggesting that knockdown of these components protected the mitochondrial folding environment. The reduced *hsp-60_pr_::gfp* expression was not simply due to a reduction in translation as separate GFP reporters under the *myo-3*, *ges-1* or ER stress-inducible *hsp-4* promoters were unaffected by the RNAi candidates (data not shown). These findings are consistent with previous experiments demonstrating that translation attenuation is protective against mitochondrial stress in yeast and *Drosophila* [12], [13]. Similarly, reduced translation has been associated with longevity and stress resistance in *C. elegans*. For example, knockdown of the *C. elegans* target of rapamycin ortholog (TOR), *CeTor*, which regulates mRNA translation in response to nutrient cues [31], or knockdown of *rsks-1*, the ribosomal S6 kinase, slows development and extends lifespan in *C. elegans* [16]. Because the long-lived *clk-1(qm30)* mitochondrial mutants have increased levels of mitochondrial stress and the stress responsive *hsp-60_pr_::gfp* reporter was specifically reduced by *CeTor* and *rsks-1*(RNAi), these findings support the hypothesis that reduced translation is beneficial to mitochondrial protein homeostasis. However, because the TOR-signaling pathway impacts many biological processes in addition to translation, other possibilities exist. Because all components identified in our RNAi screen affect protein synthesis, we sought to further characterize the role of translation attenuation in maintaining the mitochondrial protein-folding environment.

### GCN-2 Phosphorylates eIF2α in Response to Mitochondrial Stress

**Figure 3:**
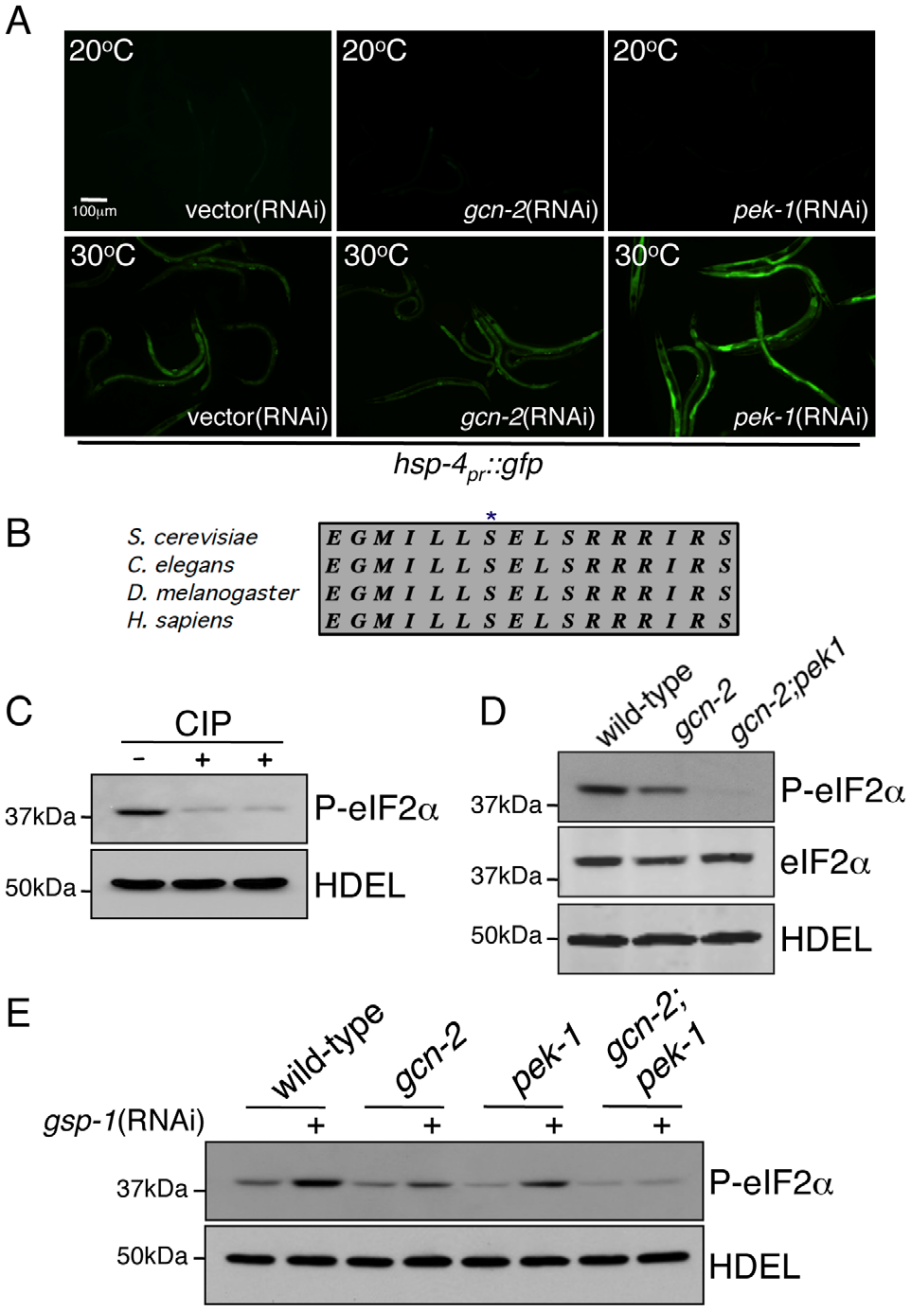
Knockdown of GCN-2 and GSP-1 Modulates eIF2α Phosphorylation Status and Mitochondrial Protein Homeostasis. (A) Fluorescent photomicrographs of *hsp-4_pr_::gfp* reporter animals raised on vector(RNAi), *gcn-2*(RNAi) or *pek-1*(RNAi). Worms were hatched on the individual RNAi plates and maintained at 20°C (upper panels) or subjected to heat shock at 30°C (3 hours) to induce ER stress (lower panels) at the L4 developmental stage. (B) Comparison of the amino acid sequence surrounding the conserved serine residue of eIF2α that is phosphorylated by the eIF2α kinases including GCN-2. (C) Immunoblot of wild-type worm lysates untreated or treated with calf intestinal phosphatase (CIP) and probed with an antibody specific to the phosphorylated form of eIF2α. The endogenous ER protein HDEL was detected with a monoclonal antibody (lower panel) and serves as a loading control. (D) Immunoblot of phosphorylated eIF2α from wild-type, *gcn-2(ok871)* and *gcn-2(ok871);pek-1(zcdf2)* animals. The anti-eIF2α and anti-HDEL immunoblots serve as loading controls. (E) Immunoblot of phosphorylated eIF2α from wild-type, *gcn-2(ok871)*, *pek-1(zcdf2)* and *gcn-2(ok871);pek-1(zcdf2)* animals raised on vector or *gsp-1*(RNAi). The anti-HDEL immunoblot serves as a loading control. Animals were raised from eggs on vector or *gsp-1*(RNAi) and harvested at the L4 stage.

In addition to *CeTor*, *rsks-1* and *cel-1*, we identified components that are known to regulate translation initiation by modulating the phosphorylation status of the translation initiation factor eIF2α. RNAi-knockdown of the eIF2α kinase **G**eneral **C**ontrol **N**on-derepressible-**2** (GCN-2) further increased *hsp-60_pr_::gfp* expression in *clk-1(qm30)* animals, suggesting a role for GCN-2 in promoting mitochondrial protein homeostasis or function (Figure 2A and 2B). The effect of *gcn-2*(RNAi) on *hsp-60_pr_::gfp* expression was not due to direct effects on GFP translation as *gcn-2*(RNAi) did not cause induction of the ER stress reporter *hsp-4_pr_::gfp* (Figure 3A) suggesting a specific role for GCN-2 in promoting mitochondrial protein homeostasis. In unstressed animals, *gcn-2*(RNAi) did not effect *hsp-60_pr_::gfp* expression, suggesting its primary role is during stress (Figure 2B). Contrary to *gcn-2*(RNAi), our RNAi screen identified *gsp-1*(RNAi), which reduced *hsp-60_pr_::gfp* expression in both the *clk-1(qm30)* and *isp-1(qm150)* strains (Figure 2A, 2B and Figure S2B). GSP-1 encodes a protein phosphatase (PP1) required for numerous cellular dephosphorylation events [32], [33] and is homologous to the yeast phosphatase required for eIF2α dephosphorylation [34].

To determine if GCN-2 and GSP-1 regulate eIF2α phosphorylation in *C. elegans*, we examined the phosphorylation status of eIF2α in whole worm lysates. We utilized an antibody that specifically recognizes the highly conserved serine that is phosphorylated by the repertoire of eIF2α kinases (S51 in mammals and S49 in *C. elegans* (Figure 3B)). Consistent with previous reports, we detected phosphorylated eIF2α in otherwise unstressed worms (Figure 3C and 3D) [35], [36], [37], which was reduced when the lysate was incubated with calf-intestine phosphatase (CIP), confirming the specificity of the antibody for the phosphorylated form of eIF2α (Figure 3C). Furthermore, in a deletion mutant lacking 1482 bases of *gcn-2* (*gcn-2(ok871)*), the level of steady-state phospho-eIF2α was reduced relative to wild-type worms (Figure 3D). In *C. elegans*, the only other known eIF2α kinase is PEK-1 (homologous to mammalian PERK [38]). Indeed, phospho-eIF2α was further reduced relative to levels of total eIF2α protein and mRNA in a strain lacking both kinases (Figure 3D and Figure S3B), further supporting the specificity of the phospho-eIF2α antibody and demonstrating the contribution of both kinases to steady state levels of eIF2α phosphorylation.

In contrast to inhibition of GCN-2 and PEK-1, GSP-1 knockdown resulted in increased levels of phospho-eIF2α consistent with it acting as a constitutive eIF2α phosphatase (Figure 3E). In either the *gcn-2(ok871)* or *pek-1(zcdf2)* deletion strains fed *gsp-1*(RNAi) there was still an increase in steady state levels of eIF2α phosphorylation likely reflecting the ability of both kinases to constitutively phosphorylate eIF2α in the absence of exogenous stress (Figure 3E). As increased phospho-eIF2α results in reduced cytosolic translation [5], [39], these data suggest that *gsp-1*(RNAi) reduces *hsp-60_pr_::gfp* induction through attenuation of cytosolic translation, thus reducing the load on the mitochondrial protein folding machinery similar to eIF2α phosphorylation and translation attenuation in the UPR^ER^ [5], [6].

**Figure 4:**
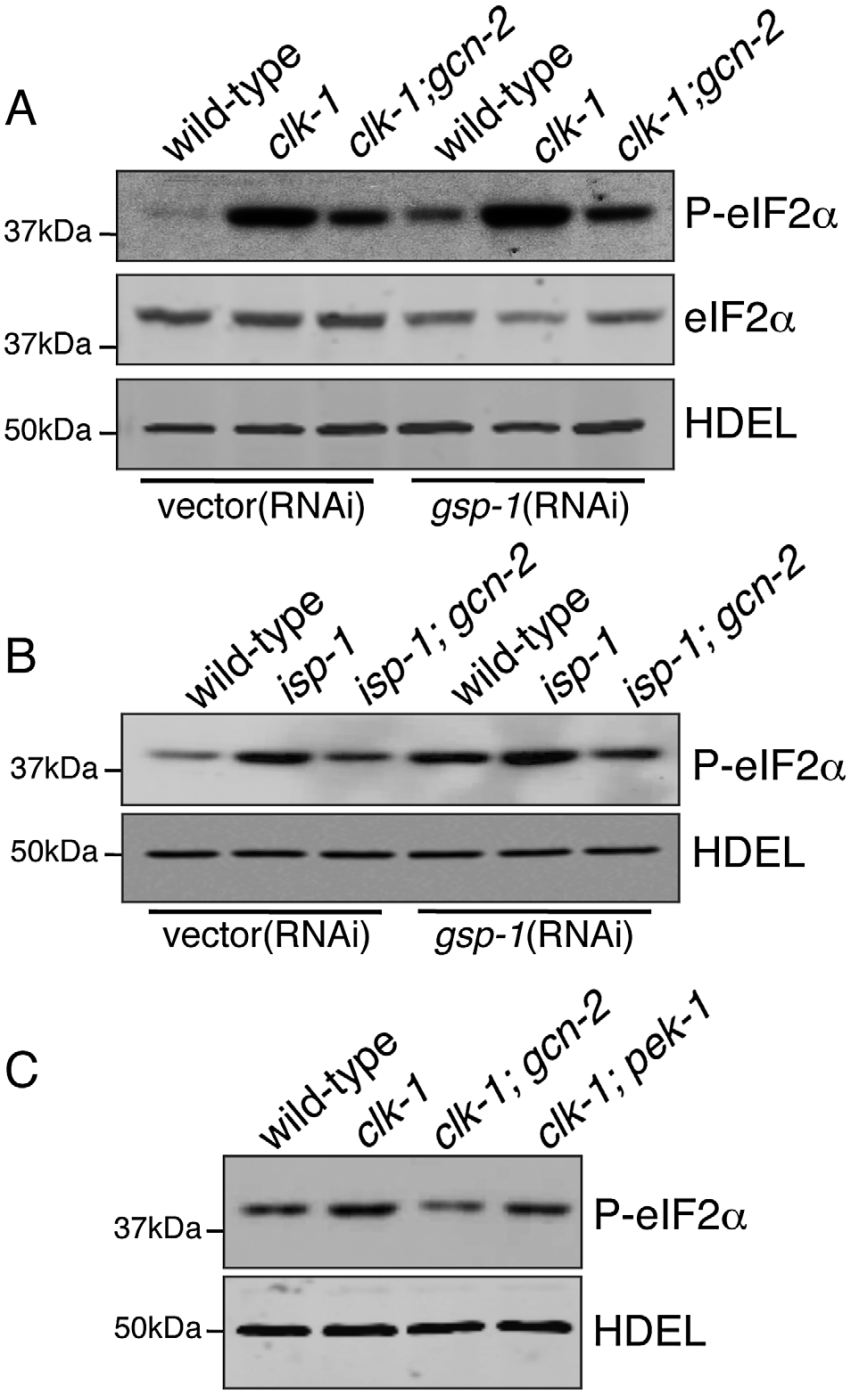
Phosphorylation of eIF2α during Mitochondrial Stress Requires GCN-2. (A) Immunoblot of phospho-eIF2α from wild-type, *clk-1(qm30)* and *clk-1(qm30);gcn-2(ok871)* animals fed vector or *gsp-1*(RNAi). The total eIF2α and anti-HDEL immunoblots serve as loading controls. Synchronized animals were raised from eggs and harvested at the L4 stage. (B) Immunoblot of phospho-eIF2α from wild-type, *isp-1(qm150)* and *isp-1(qm150);gcn-2(ok871)* animals fed vector or *gsp-1*(RNAi). The anti-HDEL immunoblot serves as a loading control. Synchronized animals were raised from eggs on the indicated RNAi plates and harvested at the L4 stage. (C) Immunoblot of phospho-eIF2α from wild-type, *clk-1(qm30)*, *clk-1(qm30)*;*gcn-2(ok871)* or *clk-1(qm30)*;*pek-1(zcdf2)* worms. The anti-HDEL immunoblot serves as a loading control. Synchronized animals were raised from eggs on vector(RNAi) plates and harvested at the L4 stage.

The data presented above suggest that GCN-2 activity promotes mitochondrial protein folding during mitochondrial stress. Therefore, we hypothesized that eIF2α phosphorylation would increase in a GCN-2-dependent manner in response to mitochondrial dysfunction. Indeed, phospho-eIF2α levels were increased relative to total eIF2α protein levels in the *clk-1(qm30)*mutant, which was absent in the *gcn-2(ok871)* mutant strain (Figure 4A). In contrast, *gsp-1*(RNAi) caused a further increase in phospho-eIF2α levels (Figure 4A). A similar result was observed in the *isp-1(qm150)* mutant, supporting the role of GCN-2 in eIF2α phosphorylation in response to stress (Figure 4B). As *gcn-2*(RNAi) perturbs the mitochondrial protein folding environment and GSP-1 knockdown promotes mitochondrial protein homeostasis as indicated by reduced *hsp-60_pr_::gfp* expression (Figure 2B and Figure S2B), these data suggest a correlation between an increase in phospho-eIF2α and a more favorable mitochondrial protein-folding environment.

It should be noted that deletion or knockdown of the other *C. elegans* eIF2α kinase PEK-1 had no obvious effect on *hsp-60_pr_::gfp* induction during mitochondrial stress (data not shown). Furthermore, the increase in eIF2α phosphorylation observed in the *clk-1(qm30)* animals was not dependent on *pek-1* indicating GCN-2 is the primary eIF2α kinase involved in maintaining mitochondrial protein homeostasis (Figure 4C). In *pek-1* deletion worms, steady state levels of phospho-eIF2α were reduced (Figure 3E), however these animals still induce eIF2α phosphorylation in response to mitochondrial dysfunction supporting the specific role for GCN-2 during mitochondrial stress (Figure 4C). A similar relationship has been described with PEK-1 and the induction of ER chaperones during ER stress. PEK-1 is specifically activated during ER stress and animals lacking PEK-1 display stronger induction of ER chaperone genes including *hsp-4* during ER stress [38]. The UPR^ER^ reporter *hsp-4_pr_::gfp* is induced during mild heat stress, a condition known to activate the UPR^ER^ but not the UPR^mt^ [9], [11]. Incubation of *hsp-4_pr_::gfp* animals at 30°C for 3 hours mildly induced GFP expression (Figure 3A and Figure S3A). However, worms raised on *pek-1*(RNAi) displayed a much stronger induction of the UPR^ER^ reporter upon heat exposure consistent with PEK-1 activity protecting ER protein homeostasis [19], [38]. Unlike *pek-*1(RNAi), *gcn-2*(RNAi) had no impact on *hsp-4_pr_::gfp* during heat stress. These results indicate that the effect of *gcn-2*(RNAi) on *hsp-60_pr_::gfp* induction was not due to dysregulation of global translation further supporting a mitochondrial stress-specific role for GCN-2.

### Loss of GCN-2 Sensitizes Worms to Conditions That Induce Mitochondrial Stress

**Figure 5:**
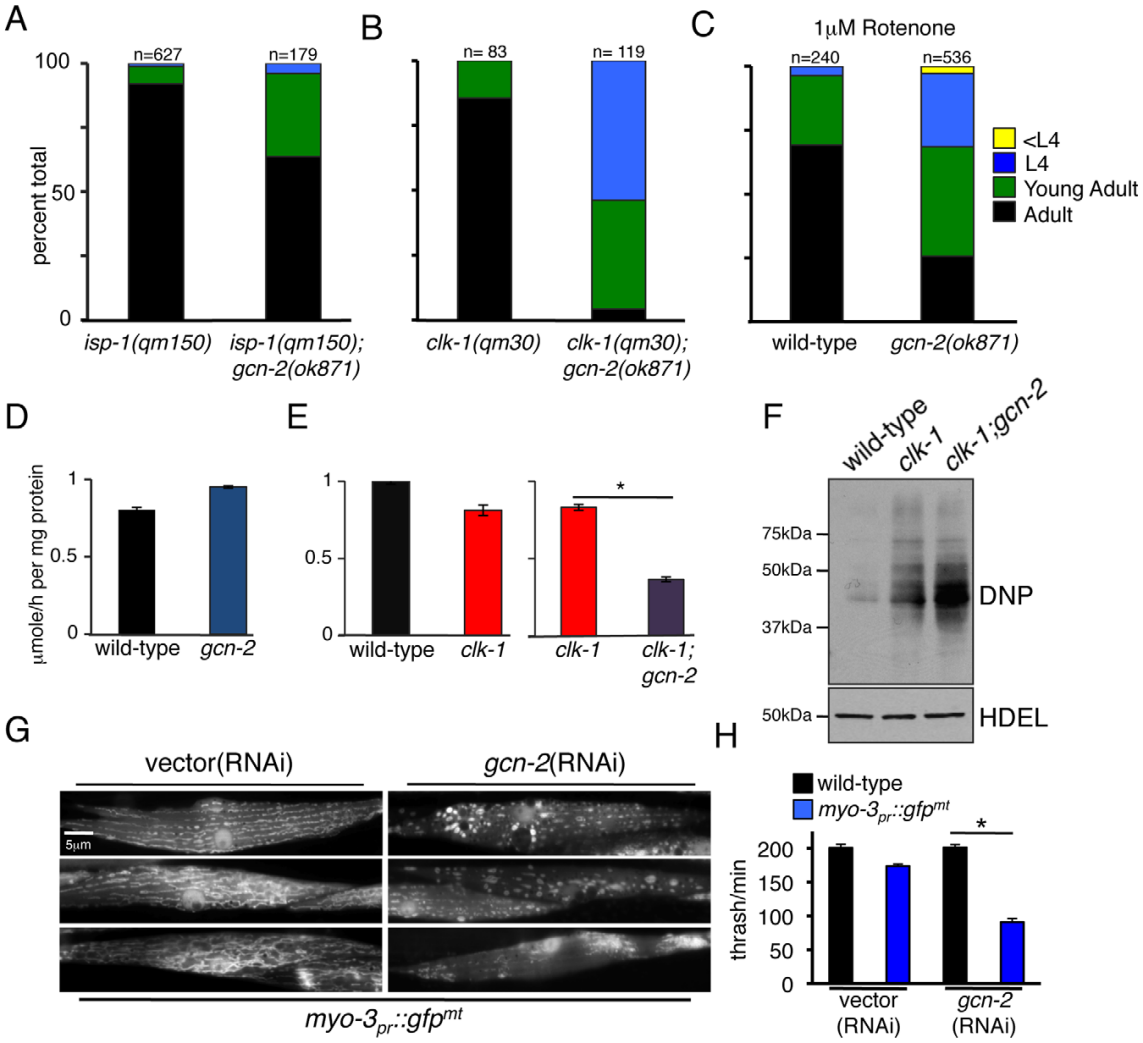
GCN-2 Is Required for Development and Mitochondrial Maintenance during Mitochondrial Stress. (A) Quantification of developmental rates of *isp-1(qm150)* and *isp-1(qm150);gcn-2(ok871)* animals. Synchronized worms were raised from eggs and animals of different developmental stages were scored and plotted as percent of total animals on day 6. (B) Developmental rates of *clk-1(qm30)* and *clk-1(qm30);gcn-2(ok871)* worms quantified as in (A) on day 5. (C) Wild-type and *gcn-2(ok871)* animals were raised on plates containing 1 µM rotenone. Rates of development were quantified on day 3. (D) Rates of oxygen consumption of synchronized wild-type or *gcn-2(ok871)* animals at the L4 stage. Shown is the mean ± SEM oxygen consumption normalized to protein content (n = 3). (E) Oxygen consumption rates of synchronized wild-type, *clk-1(qm30)* or *clk-1(qm30);gcn-2(ok871)* worms at the L4 stage. Shown is the mean ± SEM oxygen consumption normalized to protein content (n = 3, *p<0.05). (F) Immunoblots of lysates from wild-type, *clk-1(qm30)* or *clk-1(qm30);gcn-2(ok871)* probed with anti-DNP antibody (see Materials and Methods). The anti-HDEL immunoblot serves as a loading control. (G) Representative fluorescent photomicrographs of body wall muscle cells in transgenic animals expressing mitochondria-targeted GFP (*myo-3_pr_::GFP^mt^*) fed vector or *gcn-2*(RNAi). (H) Plot of the number of body strokes per minute (thrashing assay) of wild-type or *myo-3_pr_::gfp^mt^* transgenic animals raised on vector or *gcn-2*(RNAi). Shown is the mean±SEM obtained by counting strokes/min of 3-day-old animals (n = 5, *p<0.05).

As our data indicated that GCN-2 phosphorylates eIF2α in response to mitochondrial stress, we sought to determine the role of GCN-2 in development and mitochondrial maintenance during mitochondrial stress. *gcn-2* deletion or RNAi had no observable effect on worm development in the absence of stress (Figure S4). However, in the presence of mitochondrial stress caused by either the *isp-1(qm150)* or *clk-1(qm30)* mutations, *gcn-2* deletion significantly slowed development (Figure 5A and 5B). Furthermore, exposure to the NADH ubiquinone oxidoreductase (complex I) inhibitor rotenone or *spg-7*(RNAi) also significantly delayed development of *gcn-2(ok871)* worms relative to wild-type worms (Figure 5C and data not shown) indicating a protective role for GCN-2 during mitochondrial stress.

To further assess the role of GCN-2 in maintaining mitochondrial function during mitochondrial stress we examined the effect of *gcn-2* deletion on oxygen consumption in wild-type as well as mitochondrial stressed worms. We observed no difference in the rates of oxygen consumption between wild-type worms and those lacking *gcn-2* (Figure 5D), consistent with the *gcn-2(ok871)* deletion having no effect on worm development (Figure S4). *clk-1(qm30)* worms displayed a slight reduction in oxygen consumption when compared to wild-type worms consistent with mild mitochondrial dysfunction (Figure 5E) [40]. Impressively, *clk-1(qm30)* worms lacking *gcn-2* had a much lower rate of oxygen consumption than worms harboring either the *gcn-2*-deletion or *clk-1(qm30)* alone (Figure 5E), supporting a role for GCN-2 in promoting mitochondrial function during mitochondrial stress.

Elevated ROS produced by dysfunctional mitochondria can damage proteins through the formation of irreversible carobonyl modifications on lysine, cysteine, proline and threonine residues [41], [42], [43]. In order to examine levels of oxidative damage in mitochondrial stressed worms, we visualized the accumulation of carbonylated proteins using the Oxyblot system [44]. Consistent with the *clk-1(qm30)* mutation causing mitochondrial dysfunction, significantly more carbonylated material was detected in lysates from *clk-1(qm30)* worms than lysates from wild-type worms (Figure 5F). *clk-1(qm30);gcn-2(ok871)* worms displayed even more oxidative damage than worms harboring *clk-1(qm30)* alone. Because oxidative damage can perturb protein folding, these data support a role for GCN-2 in protecting the folding environment as well as mitochondrial function.

To further assess the contribution of GCN-2 in maintaining mitochondrial protein homeostasis we targeted GFP to the mitochondrial matrix via the strong muscle-specific myosin promoter (*myo-3*). High-level expression of mitochondria-targeted GFP challenges the organelle's protein folding environment by increasing the load of unfolded proteins [24], [45]. While wild-type worms were able to accommodate the increased folding load and maintain mitochondrial morphology, *myo-3_pr_::gfp^mt^* worms raised on *gcn-2*(RNAi) displayed severely perturbed mitochondrial morphology consistent with a loss of protein homeostasis and mitochondrial function [24], [46] (Figure 5G). Furthermore, in the absence of GCN-2, developmental rates (data not shown) and muscle cell function were severely reduced as determined by a motility or thrashing assay (Figure 5H). Together these data indicate that GCN-2 protects mitochondrial function during increased load of mitochondrial unfolded proteins.

### GCN-2 Is Required for the Lifespan Extension Associated with Mitochondrial Dysfunction

*clk-1(qm30)* and *isp-1(qm150)* animals, which activate ATFS-1-dependent *hsp-60_pr_::gfp* expression and GCN-2-dependent eIF2α phosphorylation, are among the numerous *C. elegans* mitochondrial mutants that exhibit lifespan extension [47], [48], [49]. It was recently reported that *ubl-5*, a small ubiquitin-like protein required for UPR^mt^ signaling [45], was required for lifespan extension in several mitochondrial mutants highlighting the importance of maintaining mitochondrial protein homeostasis [50]. Consistent with these studies, knockdown of *ubl-5* prevented *hsp-60_pr_::gfp* induction in the long-lived *clk-1(qm30)* worms (Figure S5) similar to *atfs-1*(RNAi).

**Figure 6:**
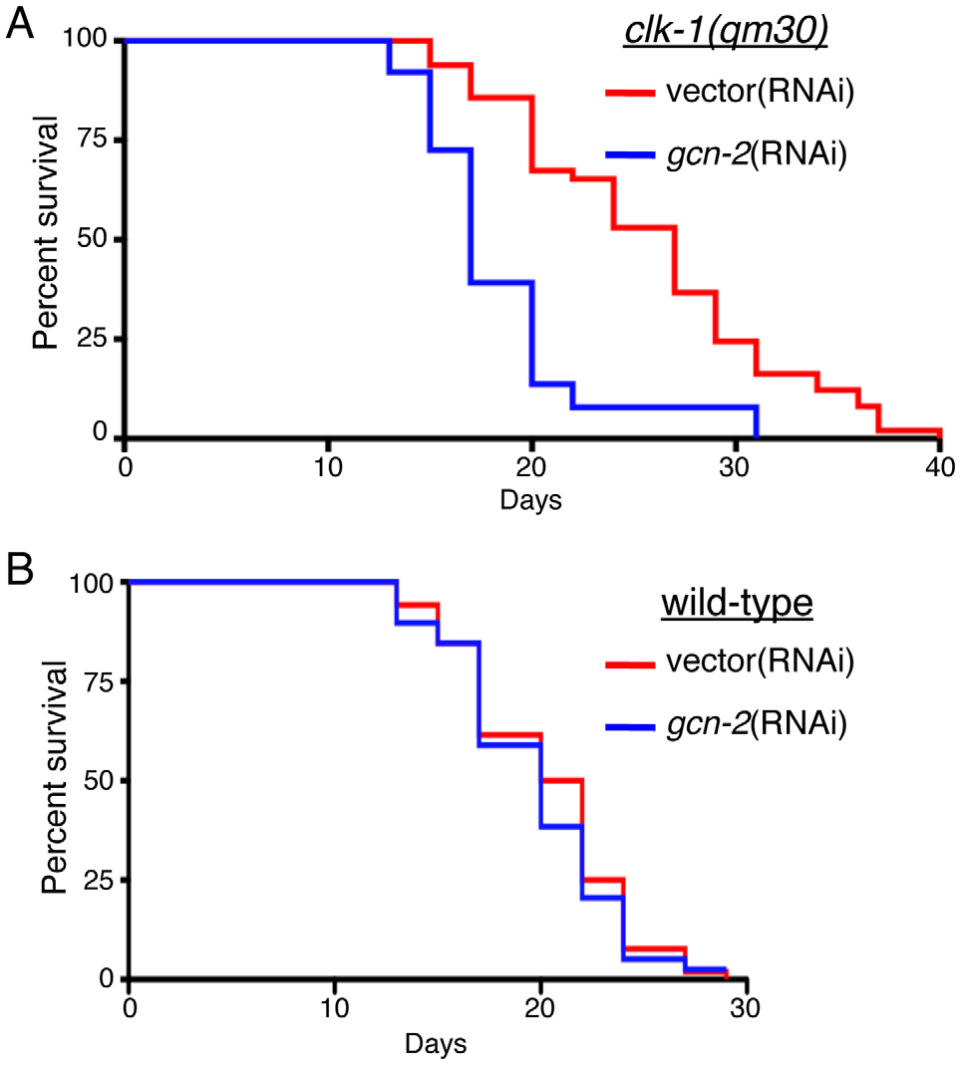
GCN-2 Is Required for the Lifespan Extension Associated with Mitochondrial Dysfunction. (A) Lifespan analysis of *clk-1(qm30)* animals fed vector (median survival 27.0 days) or *gcn-2*(RNAi) (median survival 17.0 days); p<0.0001, log-rank test. (B) Lifespan analysis of wild-type animals fed vector(RNAi) (median survival 21.0 days) or *gcn-2*(RNAi) (median survival 20.0 days); p = 0.6019 log-rank test.

Additionally, cytosolic translation attenuation also contributes to longevity in several animal models [15], [16], [17]. As GCN-2 slows cytosolic translation [51] in response to mitochondrial dysfunction, we examined the role of GCN-2 in lifespan extension associated with mitochondrial dysfunction. Interestingly, GCN-2 knockdown in *clk-1(qm30)* animals reduced their lifespan to that of wild-type worms (Figure 6A) consistent with a role for GCN-2 in lifespan extension associated with mitochondrial dysfunction. *gcn-2*(RNAi) was not generally toxic, as it did not affect lifespan or development in the absence of stress (Figure 6B and Figure S4). *gcn-2*(RNAi) also shortened the lifespan of *isp-1(qm150)* animals, but because the animals were very sick with considerable developmental defects, we were unable to determine a role for GCN-2 in longevity of these animals (data not shown). These data are consistent with GCN-2 and increased eIF2α phosphorylation contributing to the lifespan extension observed in mitochondrial mutants and further emphasizes the importance of protein homeostasis in aging.

Because *gsp-1*(RNAi) caused an increase in eIF2α phosphorylation in the absence or presence of mitochondrial stress (Figure 3E, Figure 4A and 4B), we hypothesized *gsp-1*(RNAi) would promote lifespan extension. However, the lifespan of wild-type or *clk-1(qm30)* worms on *gsp-1*(RNAi) were severely shortened (Figure S6). Interpretation of this result is complicated by the pleitropic, non-specific effects of GSP-1 knockdown. *gsp-1*(RNAi) also prevents *C. elegans* germline formation (data not shown) and is required for a variety of cellular processes including mitosis [33]. Therefore, we were unable to determine if increased eIF2α phosphorylation was sufficient to extend lifespan.

### ROS Are Required for GCN-2–Dependent eIF2α Phosphorylation during Mitochondrial Stress

**Figure 7:**
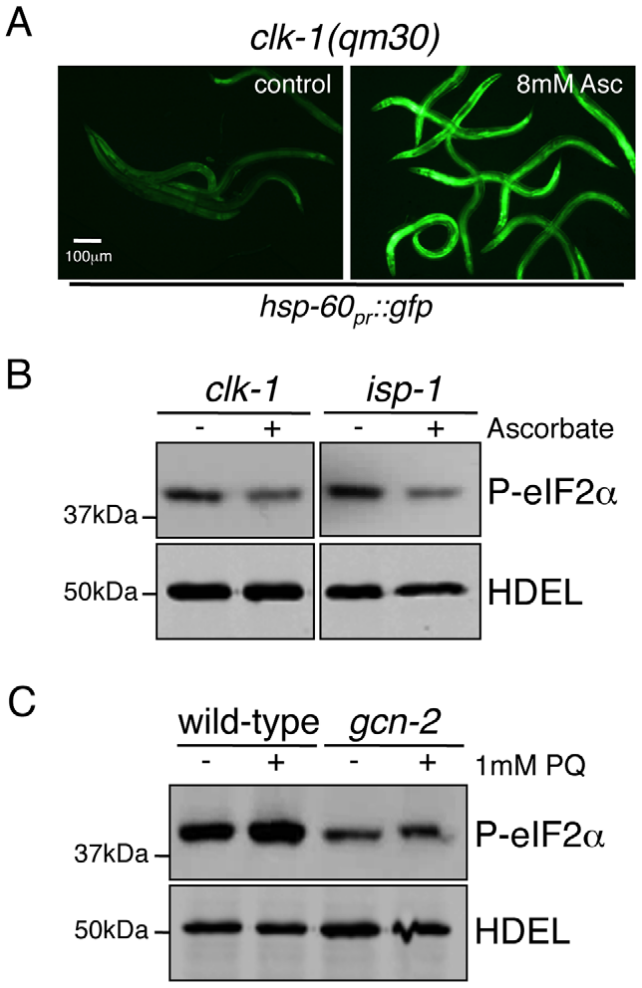
Phosphorylation of eIF2α during Mitochondrial Stress Requires ROS. (A) Fluorescent photomicrographs of *clk-1(qm30);hsp-60_pr_::gfp* animals synchronized and raised on control or plates containing 8 mM ascorbate. Images were obtained on day 5. (B) Immunoblot of phosphorylated eIF2α from *clk-1(qm30)* and *isp-1(qm150)* mutant worms untreated or treated with 25 mM ascorbate. The anti-HDEL immunoblot serves as a loading control. Worms were synchronized and allowed to develop to adulthood, at which time they were treated with 25 mM ascorbate for 16 hours prior to harvest. (C) Immunoblot of phosphorylated eIF2α from wild-type and *gcn-2(ok871)* animals treated with 1 mM paraquat (PQ). The anti-HDEL immunoblot serves as a loading control. Worms were synchronized and raised in liquid culture to the young adult stage when 1 mM PQ was added for 16 hours prior to harvest.

We next sought to determine how phospho-eIF2α status is linked to mitochondrial dysfunction. While the most well-studied mechanism of GCN-2 activation is through starvation or amino acid depletion, hydrogen peroxide exposure also stimulates GCN-2 activity through a mechanism that requires the tRNA synthetase domain [52], [53]. Because *clk-1(qm30)* and *isp-1(qm150)* worms produce increased levels of ROS (Figure 5F) that are also required for their extended longevity [22], [23], we hypothesized that ROS generated from dysfunctional mitochondria act as an upstream signaling molecule coupling mitochondrial dysfunction to GCN-2 activation. If ROS are required for the observed increase in eIF2α phosphorylation during mitochondrial stress, then treatment with ROS scavengers would phenocopy GCN-2 inhibition with respect to *hsp-60_pr_::gfp* activation and the reduced accumulation of phospho-eIF2α in the presence of mitochondrial stress. Impressively, incubation of *clk-1(qm30)* animals with the ROS scavenger ascorbate resulted in increased *hsp-60_pr_::gfp* activation, similar to *gcn-2*(RNAi) (Figure 7A and Figure 2B). Ascorbate had no effect on the induction of *hsp-60_pr_::gfp* in unstressed animals (data not shown) as observed with *gcn-2*(RNAi) (Figure 2B). We next examined the impact of ascorbate on eIF2α phosphorylation in *clk-1(qm30)* and *isp-1(qm150)* animals. Ascorbate treatment, like GCN-2 inhibition, caused a reduction of eIF2α phosphorylation in both mutants supporting a role for ROS in GCN-2 signaling during mitochondrial stress (Figure 7B).

Our data indicate a requirement for ROS in GCN-2-dependent eIF2α phosphorylation observed in response to mitochondrial dysfunction in *clk-1(qm30)* and *isp-1(qm150)* mutants. In addition to these two mutants which generate ROS, the herbicide paraquat is known to generate excessive ROS and extend *C. elegans* lifespan [22], [23]. Interestingly, similar to the *clk-1(qm30)* and *isp-1(qm150)* mutants, exposure of wild-type worms to paraquat increased eIF2α phosphorylation in a GCN-2-dependent manner (Figure 7C). In sum, these data support a protective upstream signaling role for mitochondria-generated ROS in GCN-2 activation during mitochondrial stress.

### GCN-2 Acts in a Complementary Pathway to That of ATFS-1 and Mitochondrial Chaperone Induction

**Figure 8:**
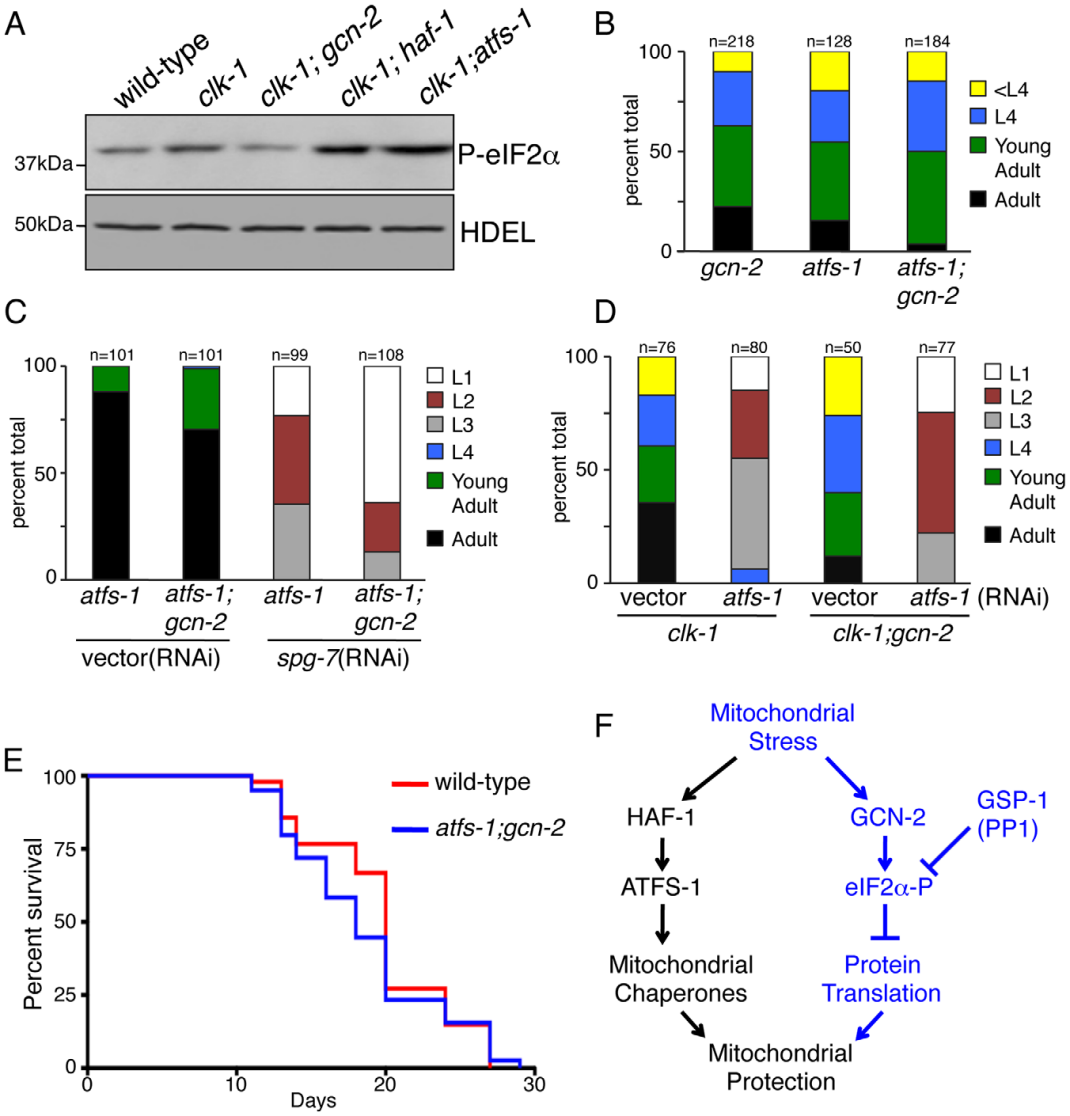
GCN-2 Acts in a Complementary Protective Pathway to that of ATFS-1 and the Induction of Mitochondrial Chaperone Genes. (A) Immunoblot of phosphorylated eIF2α from wild-type, *clk-1(qm30)*, *clk-1(qm30);gcn-2(ok871)*, *clk-1(qm30);haf-1(ok705)* animals fed vector(RNAi) and *clk-1(qm30)* animals fed *atfs-1*(RNAi). The anti-HDEL immunoblot serves as a loading control. Worms were synchronized and raised from eggs on the indicated RNAi plate and harvested at the L4 stage. (B) Quantification of developmental rates of *gcn-2(ok871)*, *atfs-1(tm4525)* and *atfs-1(tm4525);gcn-2(ok871)* animals. Synchronized worms were raised from eggs and scored as percent of total animals on day 3. (C) Quantification of developmental rates of *atfs-1(tm4525)* and *atfs-1(tm4525);gcn-2(ok871)* animals raised on vector(RNAi) or *spg-7*(RNAi). Synchronized worms were raised from eggs and scored as percent of total animals on day 3. (D) Quantification of developmental rates of *clk-1(qm30)* and *clk-1(qm30);gcn-2(ok871)* animals raised on vector(RNAi) or *atfs-1*(RNAi). Synchronized worms were raised from eggs and scored as percent of total animals on day 6. (E) Lifespan analysis of wild-type (median lifespan 20.0 days) and *atfs-1(tm4525);gcn-2(ok871)* animals (median lifespan 18.0 days); p = 0.3230, log-rank test. (F) Scheme of the hypothesized relationship of the two branches of the UPR^mt^ where HAF-1 and ATFS-1 regulate mitochondrial chaperone gene induction and GCN-2 phosphorylates eIF2α to attenuate protein translation in response to mitochondrial stress.

The above data are consistent with GCN-2-dependent eIF2α phosphorylation and translation attenuation playing a protective role in maintaining mitochondrial function similar to the protection provided by the induced mitochondrial chaperone expression regulated by HAF-1 and ATFS-1 [11]. Therefore, we sought to determine the potential interaction or relationship between GCN-2 and ATFS-1/HAF-1. If they act in complementary pathways, we hypothesized that loss-of-function of both should be more detrimental than loss of either individual pathway. Inhibition of one pathway would cause more stress placing additional burden on the other pathway to maintain the mitochondrial protein-folding environment resulting in further activation of the complementary pathway. As indicated in Figure 2B, in the presence of stress, GCN-2 inhibition results in further activation of *hsp-60_pr_::gfp* expression. Similarly, reducing eIF2α phosphorylation by inhibiting ROS accumulation resulted in increased activation of *hsp-60_pr_::gfp* activation by ATFS-1 (Figure 7A). To determine if inhibition of mitochondrial chaperone induction during stress caused a further upregulation of the GCN-2 pathway and an increase in eIF2α phosphorylation, we examined phospo-eIF2α levels in *clk-1(qm30)* animals lacking HAF-1 or ATFS-1. *clk-1(qm30)* animals displayed an increase in eIF2α phosphorylation which was further increased in combination with the *haf-1(ok705)* deletion or when fed *atfs-1*(RNAi) (Figure 8A), consistent with GCN-2 acting in a separate and complementary pathway to that of ATFS-1 and HAF-1.

We next investigated the potential synthetic interaction between GCN-2 and ATFS-1 during worm development. Either individual mutation had no obvious growth or developmental defect in the absence of stress (Figure 8B and Figure S4). However, *gcn-2(ok871);atfs-1(tm4525)* animals or the *gcn-2(ok871)* strain fed *atfs-1*(RNAi) developed somewhat slower in the absence of exogenous stress (Figure 8B and 8C). These data suggest the presence of low levels of mitochondrial stress during development that required the activity of either GCN-2 or ATFS-1.

In the presence of stress caused by *spg-7*(RNAi) or the *clk-1(qm30)* mutation, development of worms lacking *gcn-2* and *atfs-1* was severely compromised. When raised on *spg-7*(RNAi), most worms arrested at the L1 or L2 larval stage and no animals were able to reach adulthood (Figure 8C and Figure S7A). Furthermore, *clk-1(qm30)* (Figure 8D and Figure S7B) or *isp-1(qm150)* (data not shown) animals lacking both GCN-2 and ATFS-1 developed more slowly than worms lacking either individual gene. No synthetic interactions were observed between ATFS-1 and PEK-1 as *atfs-1(tm4525)* animals raised on *pek-1*(RNAi) developed at similar rates to *atfs-1(tm4525)* animals raised on a control RNAi in the absence or presence of stress (data not shown). Despite the developmental defect observed in animals lacking both ATFS-1 and GCN-2 (Figure 8B and 8C), animals lacking both genes had similar lifespans to those of wild-type worms (Figure 8E) suggesting the primary role for each pathway in the absence of exogenous stress is during development, when the majority of mitochondrial biogenesis occurs [54]. Together, these results support a model in which ATFS-1 and GCN-2 act in different yet complementary mitochondrial stress response pathways to regulate mitochondrial chaperone expression and cytosolic translation to protect mitochondrial function (Figure 8F).

## Discussion

The experiments described here implicate the eIF2α kinase GCN-2 in the maintenance of mitochondrial function and protein homeostasis. Development of worms lacking GCN-2 was impaired in the presence of mitochondrial stress which caused further induction of ATFS-1-dependent mitochondrial chaperone genes consistent with perturbed mitochondrial protein homeostasis. Furthermore, simultaneous deletion or knockdown of GCN-2 and ATFS-1 has a negative synergistic effect on animal development suggesting that GCN-2-dependent translation attenuation and ATFS-1-dependent mitochondrial chaperone gene induction act in parallel pathways to maintain mitochondrial protein homeostasis. Additionally, GCN-2 was required for development and lifespan extension in the presence of mitochondrial stress suggesting it is responsive to and protective against mitochondrial dysfunction. *gcn-2*(RNAi) or deletion inhibited eIF2α phosphorylation during mitochondrial stress. These results, along with recent experiments in yeast and flies [12], [13], support our conclusion that attenuation of cytosolic translation is protective during mitochondrial dysfunction.

Our results demonstrate that ROS generated from stressed or dysfunctional mitochondria [22], [23] are required for GCN-2-dependent eIF2α phosphorylation (Figure 7B and 7C). Furthermore, treatment with ROS inhibitors phenocopied *gcn-2*(RNAi) further exacerbating mitochondrial chaperone induction in the presence of stress (Figure 7A) suggesting ROS are required for GCN-2-dependent eIF2α phosphorylation but not ATFS-1-mediated induction of mitochondrial chaperone gene transcription. While these data support a model in which ROS act as an upstream signaling molecule, the mechanism of GCN-2 activation remains unclear. GCN-2 activation through amino acid depletion is thoroughly characterized, and requires interaction between uncharged tRNA and the tRNA synthetase domain of GCN-2 [55]. GCN-2 activation by peroxide exposure is less well understood, however it also requires the tRNA binding domain [53], [56]. Interestingly, both increased ROS and alterations in amino acid levels are known to occur in *clk-1(qm30)* and *isp-1(1qm150)* mutant worms, suggesting that ROS could participate in GCN-2 activation either through a direct interaction with the GCN-2 tRNA synthetase domain or through effects on amino acid metabolism [22], [23], [57], [58], [59]. Regardless, our results suggest a protective role for mitochondrial-generated ROS by influencing eIF2α phosphorylation, consistent with recent data indicating that low levels of ROS participate in beneficial cyto-protective stress-signaling pathways [22], [23], [60].

Attenuation of cytosolic translation slows mitochondrial import, thus reducing the folding load on mitochondrial chaperones. However, continued translation of proteins encoded by the mitochondrial genome could become detrimental when the expression of cytosolic components required for ETC complex formation is reduced. Interestingly, mitochondrial translation is tightly linked to the accumulation of imported ETC subunits and complex assembly. In their absence, mitochondrial translation is also attenuated [61]. We hypothesize that translation attenuation in the cytosol slows mitochondrial protein import leading to translational repression within mitochondria, thus reducing the overall burden on the mitochondrial protein folding and complex assembly machinery.

Protection of mitochondrial protein homeostasis and function appears to be a novel role for GCN-2 in addition to its established role during starvation [62]. The GCN-2 signaling pathway is complementary to the signaling pathway that transcriptionally upregulates mitochondrial chaperone genes during stress, which requires the mitochondrial peptide transporter HAF-1 and transcription factor ATFS-1 (Figure 8F) [11]. This parallel relationship between a reduction in organelle protein folding load and the regulation of organelle-specific protein folding machinery is similar to mechanisms that regulate ER protein homeostasis, in which another eIF2α kinase, PEK-1 (PERK in mammals), responds directly to unfolded protein stress within the ER. PEK-1-mediated translation attenuation complements the IRE-1/XBP-1 branch of the UPR^ER^, which regulates expression of ER chaperones and additional protein handling machinery [38].

In addition to a protective role during development, GCN-2 also contributes to the lifespan extension of *clk-1(qm30)* animals (Figure 6A). These mutants have disrupted mitochondrial function and elevated levels of mitochondrial chaperones (Figure 1B), consistent with a recent report that indicated a requirement for mitochondrial chaperone induction in the lifespan extension of several mitochondrial mutants [50]. Additionally, *clk-1(qm30)* animals display elevated levels of ROS that have also been shown to contribute to longevity [22], [23]. Our data support a model in which ROS and GCN-2 activate a pathway that contributes to lifespan extension, in parallel to the requirement for transcriptional induction of mitochondrial chaperone genes. The contribution of GCN-2 is most likely through cytosolic translation attenuation, which promotes stress resistance and extends lifespan in *C. elegans* [15], [16], [17].

An additional eIF2α-dependent protective activity not addressed here involves the preferential translation of mRNAs with small upstream open reading frames (uORFs). A number of uORF containing transcripts have been identified in *S. cerevisiae* [63] including the well-characterized transcription factor Gcn4 [64]. Homology searches did not reveal an obvious Gcn4 orthologue in *C. elegans*, and this avenue was not further pursued. Our RNAi screen identified components that when knocked down slow cytosolic translation as suppressors of *hsp-60_pr_::gfp* activation in stressed animals supporting a role for translation attenuation in promoting mitochondrial protein homeostasis.

Our finding that GCN-2-dependent eIF2α phosphorylation protects mitochondrial protein homeostasis raises the possibility that manipulation of phospho-eIF2α status may be a therapeutic entry point for the diverse number of degenerative diseases associated with mitochondrial dysfunction [65]. At least two strategies to accomplish this seem plausible: (1) caloric restriction to reduce cytosolic amino acid levels and activate GCN-2 to increase eIF2α phosphorylation independent of mitochondrial stress or (2) small molecule inhibition of stress-dependent eIF2α dephosphorylation to increase phospho-eIF2α levels through phosphatase inhibition [37]. It will be interesting to determine the viability of these possibilities in future studies.

## Materials and Methods

### *C. elegans* Strains and Growth Conditions

Reporter strains *hsp-60_pr_::gfp(zcIs9)V*, *myo-3_pr_::gfp^mt^(zcIs14)* and *hsp-4_pr_::gfp(zcIs4)V* have been described previously [9], [24], [45]. Where indicated, the *hsp-60_pr_::gfp(zcIs9)V* transgene was crossed into individual mutant strains of interest, with the exception of *atfs-1(tm4525)V*, which was backcrossed with N2 three times prior to crossing into the *hsp-60_pr_::gfp* background. The *clk-1(qm30)*, *isp-1(qm150)* and *gcn-2(ok871)* strains were obtained from the Caenorhabditis Genetics Center (Minneapolis, MN) and the *atfs-1(tm4525)* strain was obtained from the National BioResource Project (Tokyo, Japan). RNAi feeding experiments were performed as described [9] with constructs from the Ahringer and Vidal libraries [66], [67].

### Development and Lifespan Analysis

Worms were synchronized via bleaching and allowed to develop on the described RNAi plate or condition. For development in the presence of oxidative stress, rotenone was applied to vector(RNAi) plates and allowed to soak in prior to seeding eggs. At the time points indicated, the numbers of L1, L2, L3, L4, young adult (non-gravid) or gravid adult worms were counted on each plate and quantified as a percent of the total number of animals. For each plate, the worms in 6–8 individual fields of view were counted, and the total number combined.

For lifespan analysis, worms were synchronized as eggs and allowed to develop under the described condition for two days. At that point, 100 L4 animals were transferred to new RNAi plates and subsequently transferred to fresh plates every day for the next 5–6 days and every two days thereafter. The numbers of dead and censored worms were counted every second day for the duration of the assay [68]. Survival curves and statistical analysis were generated using Prism 5.0b software (Graphpad). Each experiment was repeated 3 times.

### Western Blots

Worms were grown under the described conditions and collected at the L4 stage for analysis as previously described [9], [24]. Phospho-eIF2α antibody (#3597S) was obtained from Cell Signaling Technology (Danvers, MA) and observed using SuperSignal West Femto Maximum Sensitivity Substrate (Thermo Scientific, Rockville, IL). GFP and HDEL immunoblots were visualized using Odyssey Infrared Imager (Li-Cor Biosciences, Lincoln, NE). Total eIF2α was assessed as described [36]. Due to limited amounts of pan-eIF2αantibody, western analysis was only performed during select experiments to confirm specificity of the phospho-eIF2α antibody in wild-type lysates and during analysis phospho-eIF2α levels in *clk-1(qm30)* animals.

For the eIF2α dephosphorylation assay, 150 µg of worm lysate was treated with calf intestinal phosphatase (CIP) for 30 minutes at 30°C prior to SDS-PAGE analysis. For ascorbate treatment, synchronized worms were grown to adulthood in liquid medium, when 25 mM ascorbate was added for 16 hours prior to western analysis.

### RNA Isolation, cDNA Synthesis, and Quantitative RT–PCR

Total RNA was isolated using RNA STAT (Tel-Test Inc, Friendswood, TX). RNA samples were prepared from the described worms at the L4 stage. cDNA was then synthesized from total RNA using a iScript cDNA Synthesis Kit (Bio-Rad Laboratories, Hercules, CA). Following mRNA isolation and cDNA synthesis, qPCR was used to determine the expression level of *eif2α* using iQ sybr green supermix and MyiQ2 Two-Color Real-Time PCR Detection System (Bio-Rad). Actin was used as a control. Fold changes in gene expression were calculated using the comparative CtΔΔCt method.

### Microscopy

Fluorescent photomicrographs were obtained using a Zeiss AxioCam MRm mounted on a Zeiss Imager.Z2 microscope or Zeiss M2 Bio stereo microscope (Carl Zeiss Imaging, Thornwood, NY).

### Oxygen Consumption and Examination of Oxidative Protein Modification

Oxygen consumption assays were performed as described [11] using a Clark type electrode [40]. To determine the accumulation of oxidative protein modifications, synchronized wild-type, *clk-1(qm30)* and *clk-1(qm30);gcn-2(ok871)* worms were harvested once they reached the L4 stage. Worm lysates were separated by SDS-Page and treated according to the Oxyblot manufacturer (Millipore, Billerica, MA).

## Supporting Information

Figure S1 Photomicrographs of *isp-1(qm150)* worms fed vector(RNAi) or *atfs-1*(RNAi) Animals were synchronized as in Figure 1C and images were obtained on day 6 after hatching.
(TIF)

Figure S2 *gcn-2*(RNAi), *CeTor*(RNAi) and *gsp-1*(RNAi) effect *hsp-60_pr_::gfp* expression in *isp-1(qm150)* worms similar to *clk-1(qm30)* worms. (A) Photomicrographs of *isp-1(qm150);hsp-60_pr_::gfp* worms raised on vector, *gcn-2* or C*eTor*(RNAi). (B) Photomicrographs of *isp-1(qm150);hsp-60_pr_::gfp* worms raised on vector or *gsp-1*(RNAi).
(TIF)

Figure S3 *gcn-2* knockdown is specific for mitochondrial dysfunction and does not effect *eif2α* mRNA levels. (A) Phase contrast images of a *hsp-4_pr_::gfp* transgenic animals raised at 20°C in top panels of Figure 3A. (B) Analysis of endogenous *eif2α* mRNA in wild-type, *gcn-2(ok871)* and *gcn-2(ok871);pek-1(zcdf2)* worms raised on vector(RNAi). Displayed is the mean +/− SEM, n = 3. In support of eIF2α protein levels in Figure 3D.
(TIF)

Figure S4 Wild-type and *gcn-2(ok871)* worms develop at similar rates in the absence of stress. Quantification of developmental rates of wild-type and *gcn-2(ok871)* animals. Synchronized worms were raised from eggs and animals of different developmental stages were scored and plotted as percent of total animals on day 3.
(TIF)

Figure S5 *ubl-5*(RNAi) inhibits *hsp-60_pr_::gfp* expression in *clk-1(qm30)* worms. Fluorescent photomicrographs of *clk-1(qm30);hsp-60_pr_::gfp* worms raised on vector or *ubl-5*(RNAi).
(TIF)

Figure S6 *gsp-1*(RNAi) dramatically shortens the lifespan of unstressed or stressed worms. Wild-type, *clk-1(qm30)* or *isp-1(qm150)* worms were synchronized on vector or *gsp-1*(RNAi). The number of worms alive was recorded on day 9 after hatching.
(TIF)

Figure S7 GCN-2 and ATFS-1 act in parallel to protect mitochondria during stress. (A) Photomicrographs of *atfs-1(tm4525)* and *atfs-1(tm4525);gcn-2(ok871)* raised on vector or *spg-7*(RNAi) as described in Figure 8C imaged at 60 or 103 hours after hatching. (B) Photomicrographs of *clk-1(qm30)* and *clk-1(qm30);gcn-2(ok871)* animals raised on vector(RNAi) or *atfs-1*(RNAi). Synchronized worms were raised from eggs on the described RNAi plate and imaged on day 8.
(TIF)
