# Supplementary figures and images for: Protective Coupling of Mitochondrial Function and Protein Synthesis via the eIF2α Kinase GCN-2
Source: PLoS Genet. 2012 Jun 14;8(6):e1002760. doi: 10.1371/journal.pgen.1002760 (PMC3375257; doi:10.1371/journal.pgen.1002760)

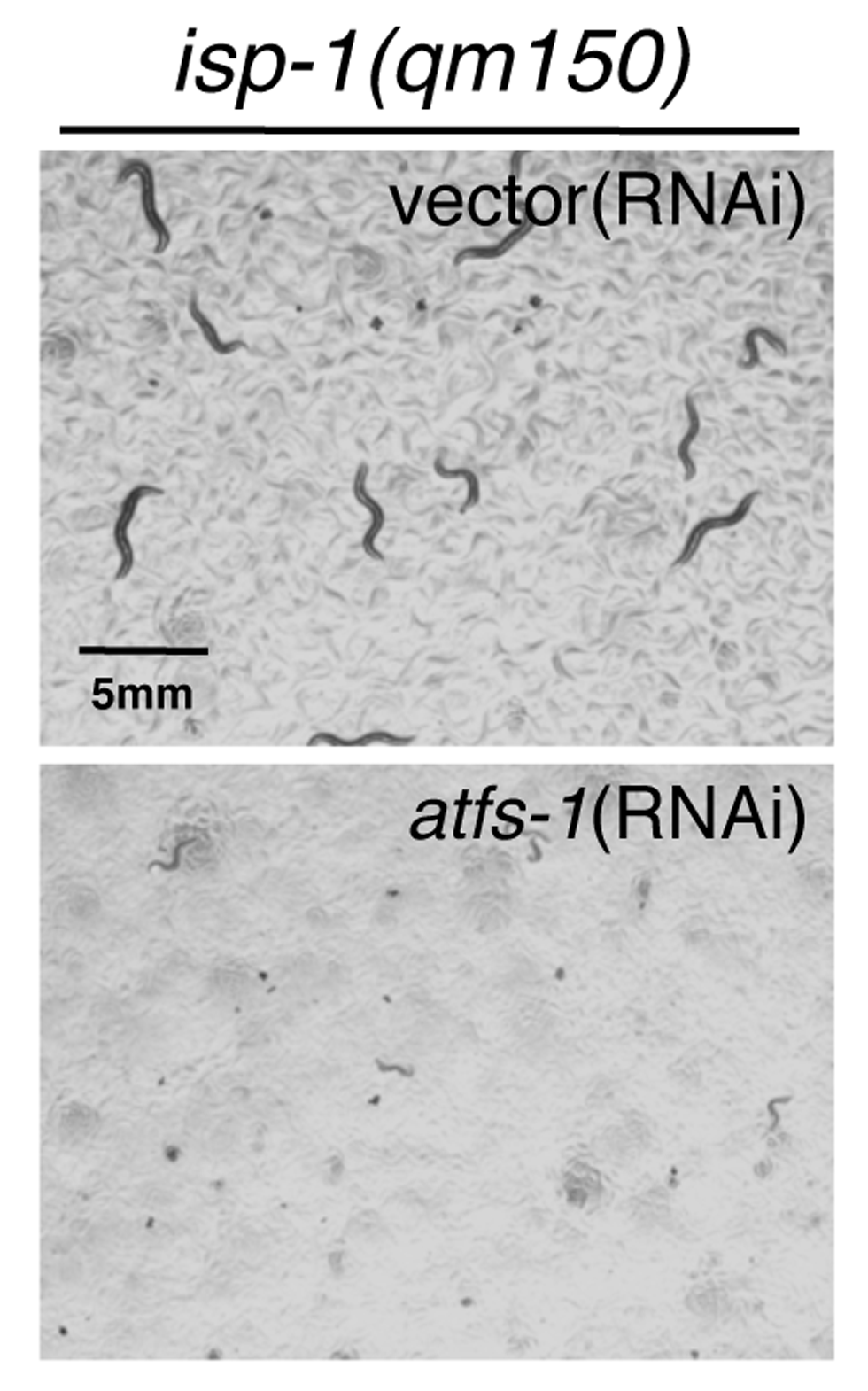

Supplement: Figure S1 — Photomicrographs of isp-1(qm150) worms fed vector(RNAi) or atfs-1(RNAi) Animals were synchronized as in Figure 1C and images were obtained on day 6 after hatching. (TIF) [file pgen.1002760.s001.tif]

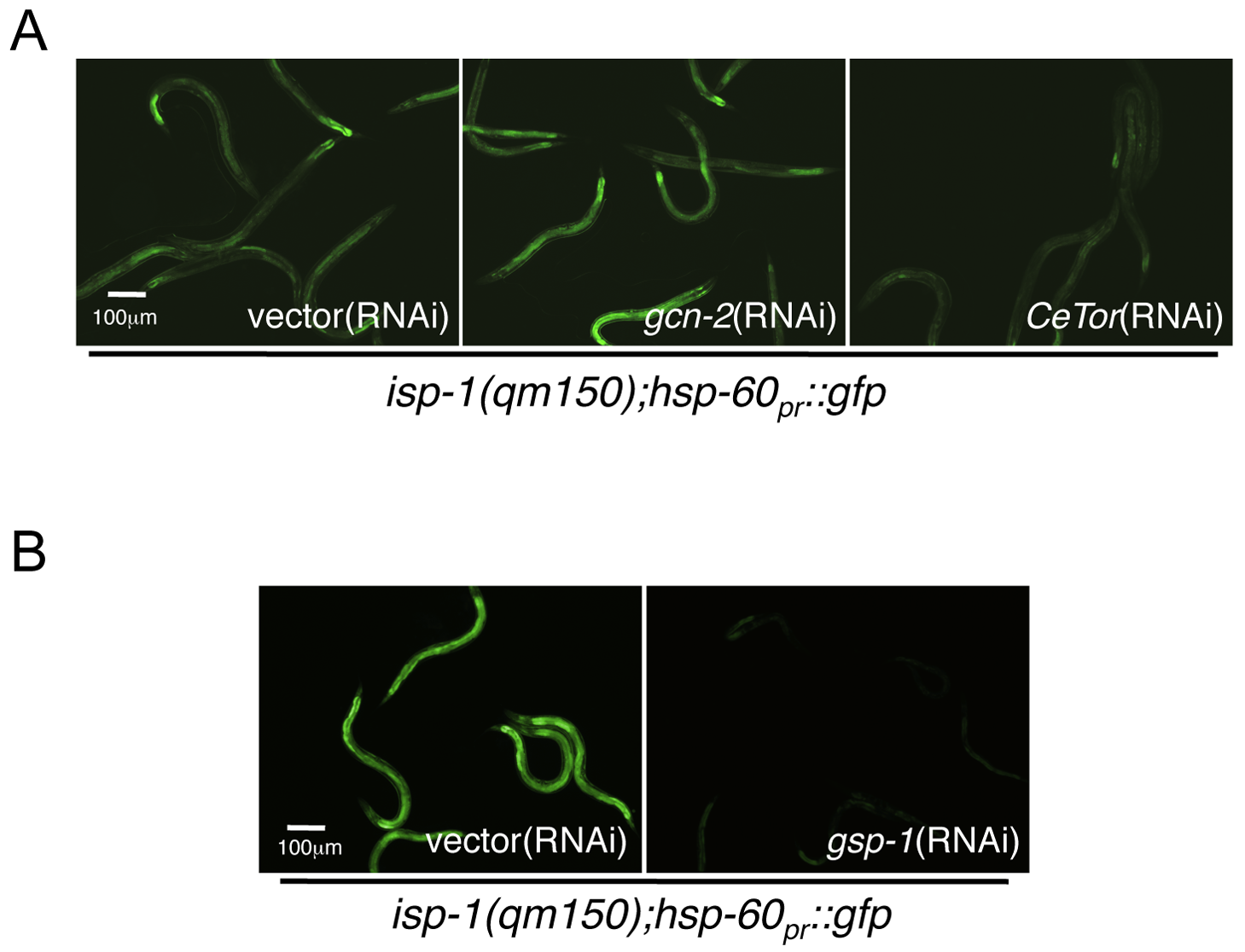

Supplement: Figure S2 — gcn-2(RNAi), CeTor(RNAi) and gsp-1(RNAi) effect hsp-60pr::gfp expression in isp-1(qm150) worms similar to clk-1(qm30) worms. (A) Photomicrographs of isp-1(qm150);hsp-60pr::gfp worms raised on vector, gcn-2 or CeTor(RNAi). (B) Photomicrographs of isp-1(qm150);hsp-60pr::gfp worms raised on vector or gsp-1(RNAi). (TIF) [file pgen.1002760.s002.tif]

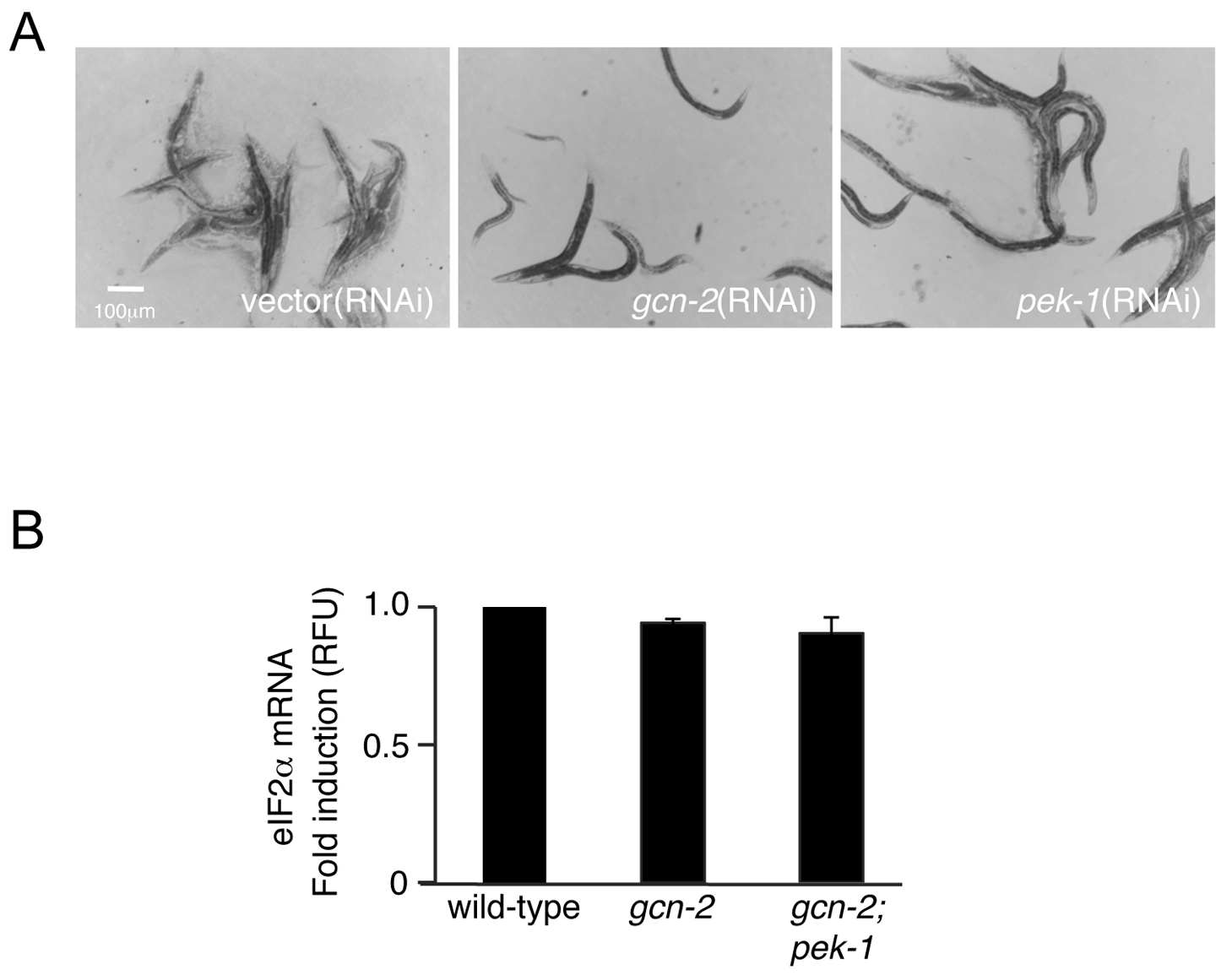

Supplement: Figure S3 — gcn-2 knockdown is specific for mitochondrial dysfunction and does not effect eif2α mRNA levels. (A) Phase contrast images of a hsp-4pr::gfp transgenic animals raised at 20°C in top panels of Figure 3A. (B) Analysis of endogenous eif2α mRNA in wild-type, gcn-2(ok871) and gcn-2(ok871);pek-1(zcdf2) worms raised on vector(RNAi). Displayed is the mean +/− SEM, n = 3. In support of eIF2α protein levels in Figure 3D. (TIF) [file pgen.1002760.s003.tif]

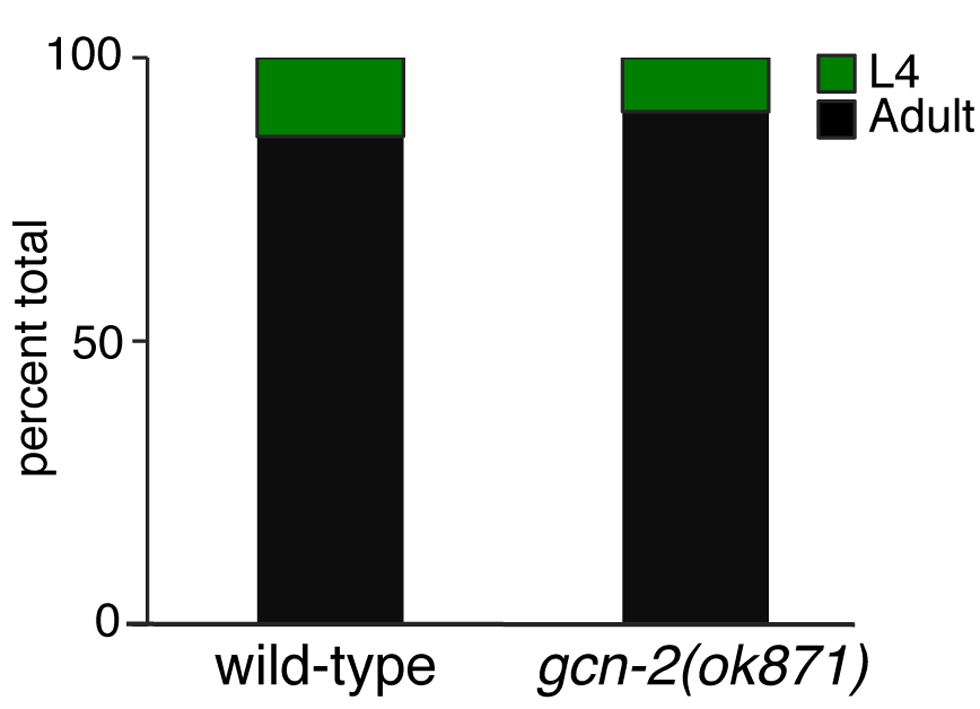

Supplement: Figure S4 — Wild-type and gcn-2(ok871) worms develop at similar rates in the absence of stress. Quantification of developmental rates of wild-type and gcn-2(ok871) animals. Synchronized worms were raised from eggs and animals of different developmental stages were scored and plotted as percent of total animals on day 3. (TIF) [file pgen.1002760.s004.tif]

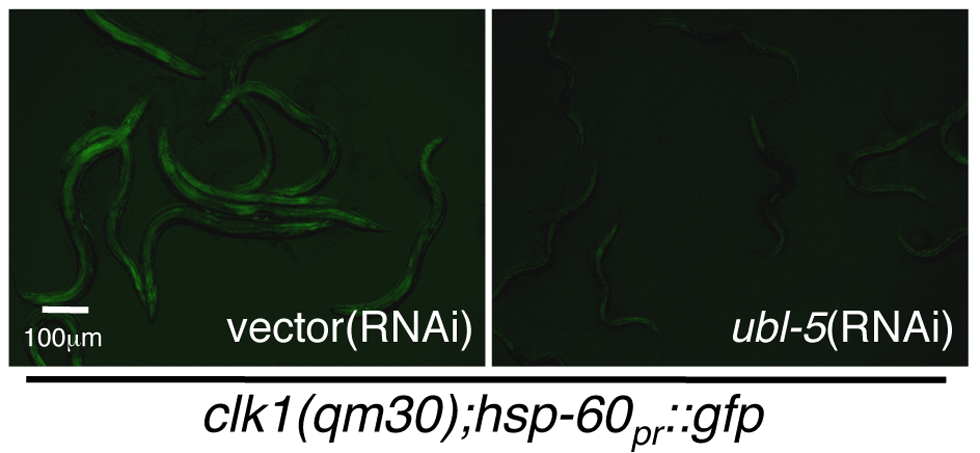

Supplement: Figure S5 — ubl-5(RNAi) inhibits hsp-60pr::gfp expression in clk-1(qm30) worms. Fluorescent photomicrographs of clk-1(qm30);hsp-60pr::gfp worms raised on vector or ubl-5(RNAi). (TIF) [file pgen.1002760.s005.tif]

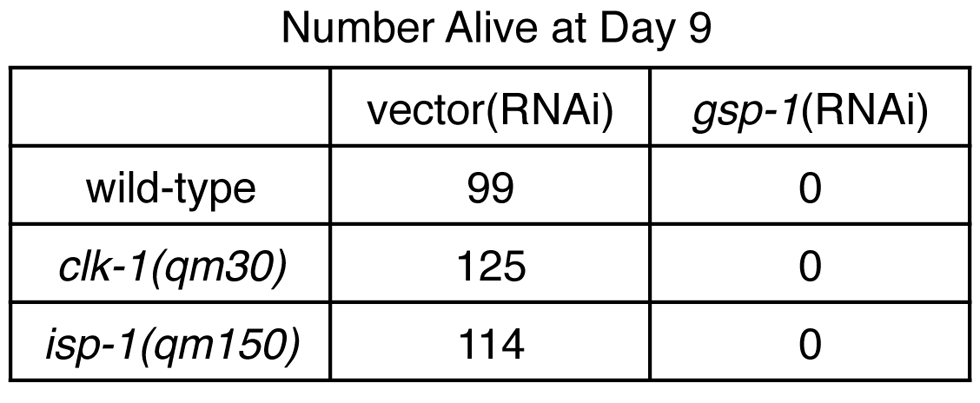

Supplement: Figure S6 — gsp-1(RNAi) dramatically shortens the lifespan of unstressed or stressed worms. Wild-type, clk-1(qm30) or isp-1(qm150) worms were synchronized on vector or gsp-1(RNAi). The number of worms alive was recorded on day 9 after hatching. (TIF) [file pgen.1002760.s006.tif]

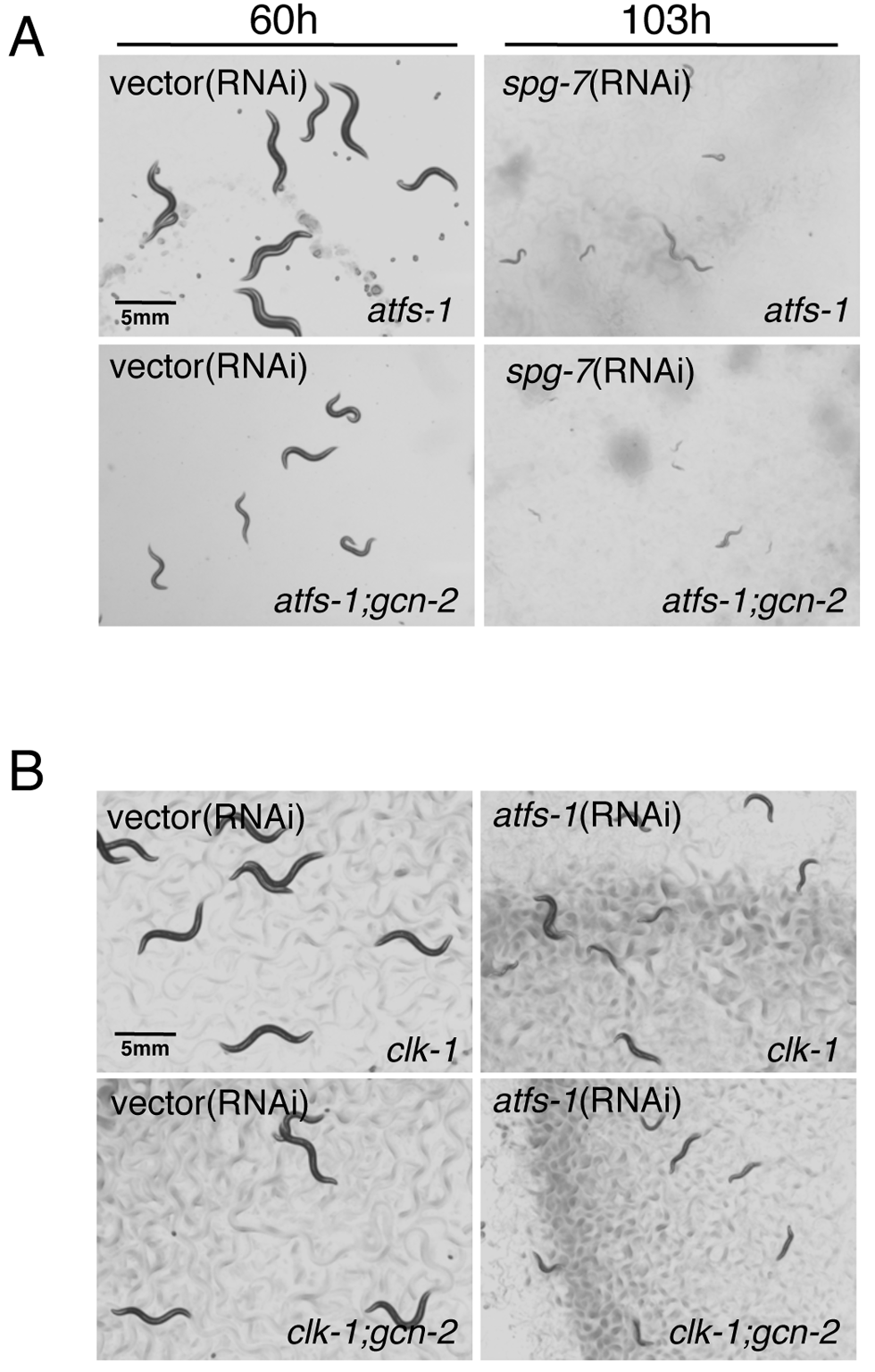

Supplement: Figure S7 — GCN-2 and ATFS-1 act in parallel to protect mitochondria during stress. (A) Photomicrographs of atfs-1(tm4525) and atfs-1(tm4525);gcn-2(ok871) raised on vector or spg-7(RNAi) as described in Figure 8C imaged at 60 or 103 hours after hatching. (B) Photomicrographs of clk-1(qm30) and clk-1(qm30);gcn-2(ok871) animals raised on vector(RNAi) or atfs-1(RNAi). Synchronized worms were raised from eggs on the described RNAi plate and imaged on day 8. (TIF) [file pgen.1002760.s007.tif]
